# A Proteomic Study of *Clavibacter Michiganensis* Subsp. *Michiganensis* Culture Supernatants

**DOI:** 10.3390/proteomes3040411

**Published:** 2015-11-12

**Authors:** Eva Hiery, Ansgar Poetsch, Tanja Moosbauer, Bushra Amin, Jörg Hofmann, Andreas Burkovski

**Affiliations:** 1 Department Biologie; Friedrich-Alexander-Universität Erlangen-Nürnberg, Staudtstr. 5, 91058 Erlangen, Germany; E-Mails: ehiery@biologie.uni-erlangen.de (E.H.); tanja.moosbauer@fau.de (T.M.); bushra.amin@fau.de (B.A.); joerg.hofmann@fau.de (J.H.); 2 Lehrstuhl für Biochemie der Pflanzen, Ruhr-Universität Bochum, Universitätsstr. 150, 44801 Bochum, Germany; E-Mail: ansgar.poetsch@ruhr-uni.bochum.de

**Keywords:** canker, *Clavibacter*, plant pathogen, secretome, wilt, xylem mimicking medium, xylem sap

## Abstract

*Clavibacter michiganensis*, subsp. *michiganensis* is a Gram-positive plant pathogen infecting tomato (*Solanum lycopersicum*). Despite a considerable economic importance due to significant losses of infected plants and fruits, knowledge about virulence factors of *C. michiganensis* subsp. *michiganensis* and host-pathogen interactions on a molecular level are rather limited. In the study presented here, the proteome of culture supernatants from *C. michiganensis* subsp. *michiganensis* NCPPB382 was analyzed. In total, 1872 proteins were identified in M9 and 1766 proteins in xylem mimicking medium. Filtration of supernatants before protein precipitation reduced these to 1276 proteins in M9 and 976 proteins in the xylem mimicking medium culture filtrate. The results obtained indicate that *C. michiganensis* subsp. *michiganensis* reacts to a sucrose- and glucose-depleted medium similar to the xylem sap by utilizing amino acids and host cell polymers as well as their degradation products, mainly peptides, amino acids and various C5 and C6 sugars. Interestingly, the bacterium expresses the previously described virulence factors Pat-1 and CelA not exclusively after host cell contact *in planta* but already in M9 minimal and xylem mimicking medium.

## 1. Introduction

The genus *Clavibacter* belongs to the group of *Actinobacteria*, and comprises a single species, *Clavibacter michiganensis*, with five established subspecies. Recently, the existence of a sixth subspecies was proposed [1]. All subspecies are plant pathogenic but differ significantly in their respective host spectrum. Despite the economic importance due to significant losses of the different crops infected, knowledge about virulence factors of *C. michiganensis* subspecies and host-pathogen interactions on a molecular level are rather limited. The best investigated subspecies in this respect is *C. michiganensis* subsp. *michiganensis*, which causes wilt and canker of tomato plants (*Solanum lycopersicum*) and bird’s eye spots on their fruits [2]. The plant is colonized either by wounds, foliar infection through stomata and trichomes or through hydatodes [3,4,5,6]. Resistant cultivars or effective chemical or antibiotic controls in field and greenhouse cultivation are unknown [7]. The infection is spread by contaminated seeds or transplants [8,9] and, consequently, testing of seeds and plant tissue as well as quarantine measurements are the most effective disease control measures.

Even in the case of *C. michiganensis* subsp. *michiganensis*, only three genes and their respective products have been studied in detail: *pat-1*, encoding a serine protease [5], *celA*, encoding a β-1,4-endoglucanase [10] and *tomA*, coding for a tomatinase which degrades the antimicrobial saponin tomatin [11]. The genes *pat-1* and *celA* are located on two natural plasmids, which are crucial for virulence and symptom development. In addition to *pat-1* and *celA*, three more putative serine proteases (*ppaJ*, *phpA* and *phpB*), which may also play an important role in pathogenicity, are encoded on these plasmids designated pCM1 and pCM2. The *tomA* gene is chromosomally encoded and located within the *chp*/*tomA* region, a putative pathogenicity island [12], which comprises also the serine protease-encoding genes *chpC*, *chpG*, *ppaA* and *ppaC*, further putative virulence factors of *C. michiganensis* subsp. *michiganensis* [13,14]. The loss of this pathogenicity island, as well as the loss of pCM1 and pCM2, results in reduced pathogenicity and symptom development in tomato [14]. Furthermore, Savidor and co-workers showed that the transcriptional regulators Vatr1 and Vatr2 are crucial for virulence in tomato [15].

When the genomes of *C. michiganensis* subsp. *michiganensis* [12] and *C. michiganensis* subsp. *sepedonicus* [16] were compared, the gene products of the *pat-1* and *celA* homologs were recognized as common virulence factors. Furthermore, a *pat-1* homolog was also observed in the genome of the closely related sugarcane pathogen *Leifsonia xyli* subsp. *xyli* [17].

The publication of the *C. michiganensis* subsp. *michiganensis* genome sequence has not only promoted bioinformatic analyses, but allowed global analysis approaches. Unfortunately, from 3080 detected genes in core genome and plasmids pCM1 and pCM2, 1030 were annotated as hypothetical, making an elucidation of their physiological function difficult [12]. Global studies might help to give at least basic information about expression of these proteins and, in fact, several studies using proteome and transcriptome analyses gave valuable information in this respect [18,19,20].

In the study presented here, the proteome of *C. michiganensis* subsp. *michiganensis* wild type strain NCPPB382382 was studied in order to identify proteins secreted to the culture supernatant. This strain carries the two natural *Clavibacter* plasmids pCM1 and pCM2, which are important for virulence [12].

## 2. Experimental Section

### 2.1. Strains and Growth Conditions

*C. michiganensis* subsp. *michiganensis* was grown at 26 to 28 °C in M9 minimal medium (5 g/L glucose, 15 g/L Na_2_HPO_4_ × 12 H_2_O, 3 g/L KH_2_PO_4_, 0,5 g/L NaCl, 1 g/L NH_4_Cl, 0.3 g/L methionine, 11 mg/L CaCl_2_, 240 mg/L MgSO_4_, 500 µg/L thiamine, 500 µg/L nicotinic acid, 0.2 mg/L FeCl_3_ × 6 H_2_O, 0.01 mg/L CuCl_2_ × 2 H_2_O, 0.01 mg/L Na_2_B_4_O_7_ × 10 H_2_O, 0.01 mg/L MnCl_2_ × 2 H_2_O, 0.01 mg/L (NH_4_)_6_Mo_7_ × 4 H_2_O, 0.04 mg/L ZnCl_2_) [21] and xylem mimicking medium XMM (1.168 g/L NaCl, 2.02 g/L KNO_3_, 1.233 g/L MgSO_4_, 0.147 g/L CaCl_2_, 22 mg/L KH_2_PO_4_, 56 mg/L K_2_HPO_4_, 1.5 µg/L FeSO_4_, 2 g/L casamino acids) [20]. For solid media, 15 g/L Bacto-agar (Oxoid, Basinstoke, UK) was added.

Bacteria were inoculated from an agar plate for overnight culture, which was used for inoculation of a new culture to an OD_600_ of 0.1–0.2. For this purpose, bacteria were harvested by centrifugation to avoid carry-over from the overnight culture. Subsequently, the cultures were incubated at 28 °C until the exponential growth phase was reached (OD_600_ approx. 0.5) [20] and further processed as described below.

### 2.2. Isolation and Filtration of Extracellular Proteins

The extraction of extracellular proteins was carried out as described for *Corynebacterium glutamicum* [22]. In short, mid-exponential phase cultures were centrifuged (25 min, 5500× *g*, 4 °C), the supernatant was collected and centrifuged again (1 h, 7000× *g*, 4 °C). To avoid proteolytic degradation, COMPLETE EDTA-free protease inhibitor cocktail was added as recommended by the supplier (Roche, Mannheim, Germany). Proteins were precipitated from the second supernatant by addition of trichloroacetic acid (10% *w*/*v* final concentration) and incubation at 4 °C (up to 3 days). Precipitated proteins were harvested by centrifugation (1 h, 8000× *g*, 4 °C), the pellet were washed first with 80% and subsequently with 100% acetone, dried on ice and resuspended in dehydration buffer (8 M urea, 50 mM Tris, 20 mM DTT, 1% sodium desoxycholate).

To improve the separation of cytoplasmic and extracellular proteins and to prevent contamination by cells and cell debris, the supernatant was filtered before precipitation using non-pyrogenic, 0.22 µm filters mounted on syringes (Sterile R, Sarstedt, Nümbrecht, Germany).

### 2.3. In-Solution Tryptic Digest

About 1.5 µg soluble proteins secreted in XMM and M9 medium were transferred to 10 KDa Hydrosart^®^ membrane filters (Sartorius Stedim biotech, Göttingen, Germany) for modified filter-aided sample preparation (FASP) [23]. Flow-through was discarded by spinning the tubes at 15,000× *g* for 20 min and proteins were reduced with 250 µL of reduction buffer containing 8 M urea, 100 mM triethylammonium bicarbonate buffer (TEAB, Sigma-Aldrich, Taufkirchen, Germany) and 20 mM dithiothreitol (DTT) for 30 min at room temperature. Alkylation of sulfhydryl groups was carried out in the same buffer containing 40 mM chloroacetamide instead of DTT for an additional 30 min in the dark. Proteins were then washed with 250 µL of 1 M urea in 50 mM TEAB and trypsinized with 1 µg of sequencing grade trypsin (Promega, Mannheim, Germany) in 1 M urea in 50 mM TEAB for 18 h at 37 °C. Peptides were extracted through centrifugation and desalted on C18 stage tips (5 min, 500× *g*). Prior to LC-MS/MS analysis, tryptic peptides were vacuum dried and resuspended in 0.1% trifluoroacetic acid (TFA).

### 2.4. NanoLC-MS/MS Analysis

Tryptic peptides of a 5 µL aliquot from each sample were analyzed by Orbitrap Fusion™ Tribrid™ mass spectrometer (Thermo Fisher Scientific, Bremen, Germany). Samples were loaded (300 µm i.d. × 5 mm, 5 μm, 100 Å) and separated (75µm i.d. × 50 cm, 3 µm, 100 Å) on PepMap100 columns using an UltiMate 3000 RSLC nano UHPLC system (Dionex, Sunnyvale, CA) with a gradient of 3%–35% system B (acetonitrile in 0.1% formic acid, system A: 1% acetonitrile in 0.05% formic acid/water) in 160 min at a flow rate of 300 nL/min at 35 °C. Eluted peptides were ionized with nanospray Flex™ ion source (Thermo Fisher Scientific) using nano-bore emitters and measured with a survey scan range of 300–2000 *m*/*z*. Most intense peptides of each scan cycle were selected and fragmented using collision-induced dissociation (normalized collision energy 35%, activation time 250 ms, isolation width 1.6 *m*/*z*). Resulting MS/MS scans were analyzed in ion trap. Dynamic exclusion was enabled for 60 sec and excluded after 1 time. Raw data files were processed against *C. michiganensis* subsp. *michiganensis* NCPPB382 database (Proteome Id: UP000001564) comprised in UniProt [24] using Peaks v7.5 (released on 16 June, 2015, Bioinformatics Solutions Inc., Waterloo, ON, Canada). Theoretical masses for peptides were generated by trypsin with a maximum of two missed cleavages and their product ions were compared to the measured spectra with following parameters: carbamidomethyl modification was set as fixed and oxidation of methionine residues and carbamidomethylated lysines were set as an optional modification. Mass tolerance was set to 10 ppm for survey scans and 0.5 Da for fragment mass measurements. Peptide charges of 2–7 were allowed. Only resulting peptides with false discovery rate (FDR) below 0.01 were regarded as identified [25,26].

Label free quantification was performed with Peaks Q (Bioinformatics Solutions Inc.) out of six samples in two groups with a FDR threshold of 1%. Samples were not normalized and proteins considered for calculation had at least two unique peptides. Proteins were only regarded as identified, when these were found in two out of three samples.

### 2.5. Analysis of Functional Categories and Localization of Proteins

Classification of proteins in functional categories was based on the lists provided by Flügel and co-workers [18]. Analysis of protein localization was carried out by two approaches, (i) manually based on the protein annotation; and (ii) automatically using the proteome discoverer version 1.14 software package (Thermo Fisher Scientific, Bremen, Germany), SignalP 4.1 and the SecretomeP 2.0 server [27,28].

## 3. Results and Discussion

The functions of the genes of *C. michiganensis* subsp. *michiganensis* were predicted before and the genes were dedicated to specific COG groups by Flügel and coworkers, who introduced a fifth COG-related group designated as “potentially relevant in the phytopathogenic interaction” [18]. Most of the predicted gene products fulfil their functions in processes like metabolism, cellular processes and information and storage. Some proteins, like proteases and hydrolytic enzymes, seem to be important for pathogenicity. First global experiments to identify genes and proteins, which are important for pathogenicity, were carried out before. In these studies, *C. michiganensis* subsp. *michiganensis* was grown *in planta* or *in vitro* in a minimal medium with 5% tomato homogenate, 10% xylem sap or in a xylem surrogate medium [18,19,20]. In the study presented here, culture supernatants of cells grown in M9 and xylem mimicking medium [20] were studied in a proteomic approach.

### 3.1. Analysis of C. michiganensis Subsp. michiganensis M9 and XMM Culture Supernatants

In this study, the extracellular proteome of *C. michiganensis* subsp. *michiganensis* was analyzed after growth in minimal medium and xylem surrogate medium. In total, 1872 proteins were identified in M9 and 1766 proteins in XMM (for a complete list, see supplementary material Table S1). Since the *C. michiganensis* subsp. *michiganensis* genome encodes only 3107 annotated genes [29], this high number can only be explained when cells lysis during growth and release proteins into the medium is considered.

**Figure 1:**
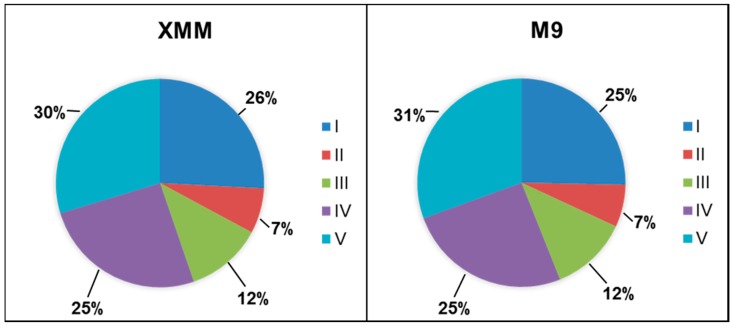
Distribution of functional categories of proteins isolated from M9 and XMM medium. COG I, metabolism; COG II, cellular processes; COG III, information storage and processing; COG IV, potentially relevant in phytopathogenic interaction; COG V, poorly characterized.

**Figure 2:**
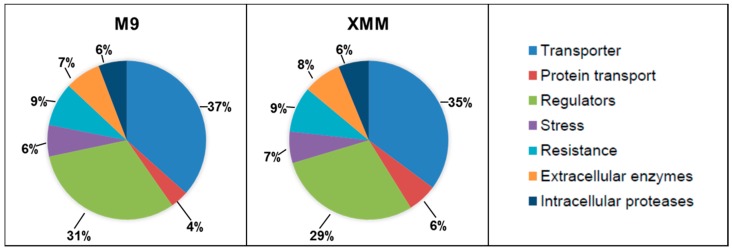
Distribution of COG IV subgroups of proteins isolated from M9 and XMM medium. For explanation of COG IV groups, see the right panel.

While 203 proteins were exclusively detected in M9 and 97 proteins in XMM, 1669 proteins were detected under both growth conditions, indicating a rather small difference in protein expression and the use of rather similar protein sets in the two media. When these proteins were classified in functional categories according to Flügel and co-workers [18], a very similar distribution was found for M9- and XMM-isolated proteins (Figure 1). Thirty percent of all detected proteins were members of COG V (poorly characterized), followed by about 25% attributed to COG I (metabolism) and COG IV (potentially relevant in the phytopathogenic interaction), respectively, and lower shares of COG III (information storage and processing) and COG II (cellular processes). When COG IV was analyzed in more detail (Figure 2), again the distribution of M9 and XMM-isolated proteins was highly similar with the majority classified as transporters, followed by regulators and smaller shares of proteins involved in protein transport, regulation, stress response, resistance and the groups of extracellular enzymes and intracellular proteases.

This result is consistent with recently carried out transcriptome analyses [20], which also emphasized the role of transport processes for *C. michiganensis* subsp. *michiganensis.*

### 3.2. Analysis of the Putative C. michiganensis Subsp. michiganensis Secretome

The detected proteins were analyzed in respect to their predicted localization according to their annotation (Table 1). Based on their annotation only thirteen proteins had a clear cell wall or surface localization and might be either actively secreted into the medium or have been sheared from the cell surface during incubation in the shaking flasks. These were putative penicillin binding proteins, putative sortases, putative cell surface proteins, a capsular polysaccharide synthesis protein, and a putative NplC/P60 protein.

In addition to these, a high number of proteins related to sugar, amino acid and peptide uptake were identified, mainly substrate-binding subunits of ABC transporters, which are located at the outside of the cytoplasmic membrane. Among these, sugar transporter subunits were the most prominent group. Fifteen not further characterized putative sugar ABC transporter substrate-binding proteins were detected in addition to a putative l-arabinose ABC transporter substrate-binding protein and a putative α-glucoside ABC transporter substrate-binding protein.

**Table 1:** Predicted functions of *C. michiganensis* subsp. *michiganensis* proteins detected in culture supernatants. Expression in the respective media is indicated by + absence by -, proteins identified only after filtration are indicated in bold.

Identifier	Annotated Function	M9/XMM
CMM_0013	putative sortase	+/+
CMM_0017	putative penicillin binding protein	+/+
CMM_0039	putative extracellular serine protease	+/+
CMM_0041	putative extracellular serine protease	+/+
CMM_0053	putative extracellular serine protease	+/+
CMM_0059	putative extracellular serine protease	+/+
**CMM_0075**	**putative extracellular serine protease**	**+/+**
CMM_0090	β-xylanase	+/+
CMM_0109	putative sugar ABC transporter substrate-binding protein	+/+
CMM_0129	putative sortase	+/−
CMM_0141	putative secreted protein	+/−
CMM_0166	putative iron-siderophore ABC transporter substrate-binding protein	+/+
**CMM_0289**	**conserved secreted/exported protein**	**+/+**
CMM_0296	putative sugar ABC transporter substrate-binding protein	+/+
**CMM_0345**	**conserved secreted/exported protein**	**+/+**
CMM_0359	putative sugar ABC transporter substrate-binding protein	+/+
CMM_0363	putative iron-siderophore ABC transporter substrate-binding protein	+/+
CMM_0423	putative sugar ABC transporter substrate-binding protein	+/+
CMM_0430	putative cell surface protein	+/+
CMM_0431	putative hemagglutinin/hemolysin-related protein	+/+
CMM_0435	putative iron-siderophore ABC transporter substrate-binding protein	+/+
CMM_0613	levanase	+/+
**CMM_0667**	**secreted phosphoesterase**	**+/+**
CMM_0706	putative penicillin binding protein	+/−
CMM_0792	putative sugar ABC transporter substrate-binding protein	+/+
CMM_0795	putative extracellular nuclease/phosphatase	+/+
CMM_0799	putative peptide ABC transporter substrate-binding protein	+/+
CMM_0819	putative cell surface protein	+/+
CMM_0825	putative cell surface protein	+/+
CMM_0827	capsular polysaccharide synthesis protein	+/+
CMM_0840	putative NplC/P60 protein	+/+
CMM_0866	putative α-glucoside ABC transporter substrate-binding protein	+/+
CMM_0879	putative sugar ABC transporter substrate-binding protein	+/+
CMM_0915	putative penicillin binding protein	+/+
CMM_0919	putative penicillin binding protein	+/+
CMM_0944	putative sugar ABC transporter substrate-binding protein	+/+
CMM_0975	putative ABC transporter substrate-binding protein	+/+
CMM_0976	putative ABC transporter substrate-binding protein	+/+
CMM_1022	putative secreted protein	+/+
**CMM_1031**	**putative secreted protein**	**+/+**
CMM_1032	putative secreted protein	+/+
CMM_1129	putative siderophore interacting protein	+/−
CMM_1243	putative sugar ABC transporter substrate-binding protein	+/+
**CMM_1250**	**putative secreted protein**	**+/+**
CMM_1262	putative sugar ABC transporter substrate-binding protein	+/+
CMM_1304	conserved secreted protein	−/+
CMM_1314	putative iron ABC transporter substrate binding protein	+/+
**CMM_1389**	**conserved secreted/exported proteins**	**+/+**
CMM_1405	conserved secreted protein	+/+
CMM_1406	putative secreted protein	+/+
CMM_1411	putative cell surface protein	+/+
CMM_1450	putative secreted protein	+/+
CMM_1478	putative peptide ABC transporter substrate-binding protein	+/+
CMM_1532	putative proline/glycine/betaine/choline ABC transporter substrate-binding protein	+/+
CMM_1557	putative secreted protein	+/+
CMM_1673	β-xylanase	+/+
CMM_1674	β-xylanase	+/+
CMM_1790	putative anion ABC transporter substrate-binding protein	+/+
CMM_1865	putative penicillin binding protein	+/+
CMM_1947	putative extracellular serine protease	+/+
CMM_1948	putative extracellular serine protease	+/+
CMM_1960	putative peptide ABC transporter substrate-binding protein	+/+
CMM_2106	putative sugar ABC transporter substrate-binding protein	+/+
CMM_2169	putative RTX toxin	+/+
CMM_2176	conserved secreted lipoprotein	+/+
CMM_2178	putative secreted protein	+/+
CMM_2180	putative peptide ABC transporter substrate-binding protein	+/+
CMM_2185	putative peptide ABC transporter substrate-binding protein	+/+
CMM_2238	putative sugar ABC transporter substrate-binding protein	−/+
CMM_2283	putative metal ABC transporter substrate-binding protein	+/+
CMM_2349	putative iron-siderophore ABC transporter substrate-binding protein	+/+
CMM_2410	putative sugar ABC transporter substrate-binding protein	+/+
CMM_2434	putative extracellular serine protease	+/+
CMM_2438	putative L-arabinose ABC transporter substrate-binding protein	+/+
CMM_2467	putative secreted hydrolase	+/+
CMM_2505	phosphate binding protein PstS	+/+
CMM_2566	putative branched-chain amino acid ABC transporter substrate-binding protein	+/+
CMM_2628	putative polar amino acid ABC transporter substrate-binding protein	+/+
**CMM_2676**	**putative β-lactamase**	**+/+**
CMM_2699	putative sugar ABC transporter substrate-binding protein	+/+
CMM_2733	putative sugar ABC transporter substrate-binding protein	+/+
CMM_2842	putative sugar ABC transporter substrate-binding protein	+/+
CMM_2941	putative metal ABC transporter substrate-binding protein	+/+
**pCM1_0018**	**putative secreted protein**	**+/+**
**pCM1_0020**	**CelA**	**+/+**
pCM1_0023	putative extracellular serine protease	
pCM2_0025	putative secreted protein	+/+
pCM2_0028	conserved secreted protein	+/+
pCM2_0053	putative extracellular serine protease	+/+
**pCM2_0054**	Pat-1	+/+

Slightly less abundant were amino acid and peptide uptake components with a putative polar amino acid ABC transporter substrate-binding protein, a putative branched-chain amino acid ABC transporter substrate-binding protein, a putative proline/glycine/betaine/choline ABC transporter substrate-binding protein, and five putative peptide ABC transporter substrate-binding proteins.

The third major group of proteins were related to the uptake of iron and other ions; namely, a putative siderophore interacting protein, four putative iron-siderophore ABC transporter substrate-binding proteins, a putative iron ABC transporter substrate binding protein, a putative anion ABC transporter substrate-binding protein, two putative metal ABC transporter substrate-binding proteins, and the phosphate binding protein PstS.

Additionally, two putative ABC transporter substrate-binding protein without further annotation were found.

Twenty-three proteins were annotated to be secreted. Nine putative secreted proteins, three conserved secreted proteins, nine putative extracellular serine proteases, a conserved secreted lipoprotein, a putative secreted hydrolase. Furthermore, five polymer degrading enzymes were found; namely a levanase, three β-xylanases, and a putative extracellular nuclease/phosphatase, in addition to two proteins, which might be involved in host interaction, a putative RTX toxin and a putative hemagglutinin/hemolysin-related protein.

From these protein, the putative siderophore interacting protein CMM_1129, the putative penicillin binding protein CMM_0706, the putative sortase CMM_0129, and the putative secreted protein CMM_0141 were exclusively found in M9 supernatants, while the putative sugar ABC transporter substrate binding protein CMM_2238 and the conserved secreted protein CMM_1304 were only detected after XMM incubation.

In summary, *C. michiganensis* subsp. *michiganensis* grown in either standard M9 minimal medium or xylem-mimicking medium XMM secrete a cocktail of proteases and other polymer degrading enzymes, while a wide variety of transporters is expressed in parallel to take up the degradation products.

### 3.3. Effect of Filtration on the Detectable Secretome

As described before, application of 0.22 µm filters might improve enrichment of secreted protein. In case of xylem sap filtration, the number of proteins was reduced from 1013 to 165 after introduction of a filtration step [19]. Unexpectedly, the effect was less drastic in this study. In total 1276 proteins were detected in the M9 culture filtrate and 976 proteins in the XMM culture filtrate, which results in a reduction of detectable proteins by only one third, while Savidor and co-workers found a massive reduction of 84% [19]. Nevertheless, also in this study an enrichment of secreted proteins was observed even by simple inspection of protein lists. In the filtrate—but not in the unfiltered samples—the following secreted proteins were found: three putative secreted proteins, three conserved secreted/exported proteins, a secreted phosphoesterase, a putative β-lactamase, and a putative extracellular serine protease. Moreover, the previously described virulence factors Pat-1 and CelA were identified now both, in M9 and XMM supernatants (Table 1). The expression of these virulence factors in minimal media was already observed in previous studies [18,20,21], indicating that plant contact is not crucial for an induction of virulence genes.

In parallel to the data analysis described above, bioinformatics analyses were carried out. More than 76% of the proteins identified in M9 and XMM filtrates had no annotation, and only about 0.4% were identified as extracellular or cell surface proteins by the proteome discoverer. Further analyses of proteins differentially expressed proteins in M9 and XMM medium using Peaks 7.5 (Bioinformatics Solution Inc., Canada), SignalP and Secretome 2.0 revealed 4 proteins with classical signal sequence and 10 non-classically secreted proteins (Table 2). Half of these proteins lacked an annotated function. However, Pat-1 and a putative extracellular serine protease, which might also contribute to virulence were more abundant in XMM.

**Table 2:** Differentially expressed proteins in filtered culture supernatants.

Identifier	Annotated Function	Upregulated in
Proteins with classical signal peptide		
CMM_0166	putative Fe^3+^ siderophore	M9
pCM2_0042	uncharacterized protein	XMM
pCM2_0054	Pat-1	XMM
CMM_0052	putative extracellular serine protease	XMM
Non-classically secreted proteins		
CMM_1654	uncharacterized protein	M9
CMM_1841	putative cytochrome c oxidase	M9
CMM_2064	uncharacterized protein	M9
CMM_2634	uncharacterized protein	XMM
CMM_1793	elongation factor P	M9
pCM2_0042	uncharacterized protein	XMM
CMM_0166	putative Fe^3+^ siderophore	M9
CMM_0486	uncharacterized protein	M9
CMM_0517	putative transcriptional regulator	M9
CMM_0581	uncharacterized protein	M9

## 4. Conclusions

*C. michiganensis* subsp. *michiganensis* is an important tomato pathogen, which is, however, hardly studied. Only 116 entries for the query “*Clavibacter michiganensis* subsp. *michiganensis*” were found in a PubMed search end of October 2015. Proteome studies might help to improve the understanding of this pathogen. As shown in this study, bacteria grown in either standard M9 minimal medium or xylem-mimicking medium XMM secrete a cocktail of proteases and other polymer degrading enzymes into the surrounding, while a wide variety of transporters is expressed in parallel to take up the degradation products, especially sugars and amino acids. *In planta*, this will lead to efficient utilization of xylem sap components, mainly amino acids [20], and host cell polymer degradation products, *i.e.*, amino acids, peptides, pentoses and hexoses. In fact, Savidor and co-workers found different proteases (ChpE, Pat-1, PpaC, PpaH, SbtC), the cellulose CelA, a pectate lyase and a polygalacturonidase in filtered xylem sap of *C. michiganensis* subsp. *michiganensis*-infected tomato plants [19].

Supernatants of minimal medium cultures were significantly contaminated with cytoplasmic proteins in this study, despite the fact that carry-over from the inoculum was avoided and exponential growth phase cells were used. In contrast to previous work [19], filtration of supernatants had only a relatively small effect, indicating a considerable amount of cell lysis during growth of *C. michiganensis* subsp. *michiganensis* in shaking flasks, which was unexpected due to the rigid Gram-positive cell wall structure of this species. The reason for this observation has to be investigated in future studies.

Nevertheless, filtration of culture supernatants led to an enrichment of secreted proteins and under these conditions, the known major virulence factors Pat-1 and CelA were identified as well. The identification of these and other virulence factors indicate that *C. michiganensis* subsp. *michiganensis* expresses virulence determinants not exclusively after host cell contact *in planta* but already in minimal medium, *i.e.*, in an *in vitro* situation.

In summary, proteome studies are a valuable tool to understand physiology and adaptations of the *C. michiganensis* subsp. *Michiganensis*, however, these studies are hampered by the high number of proteins lacking an annotated function and the overall very limited knowledge about this bacterium. Especially studies on protein export in *C. michiganensis* subsp. *michiganensis* would be interesting not only in respect to this study but also due to the fact that effector proteins might be secreted into the host to manipulate plant metabolism and pathogen defense.

## Supplementary Materials

Supplementary File 1
